# Severe microscopic polyangiitis with unilateral vocal cord paralysis as initial manifestation

**Published:** 2017-03-30

**Authors:** Luis Felipe Flores-Suárez, Marco Antonio Alba, Gabriel Tona

**Affiliations:** Instituto Nacional de Enfermedades Respiratorias, Ciudad Mexico , Mexico.

**Keywords:** Microscopic polyangiitis, vocal cord paralysis, larynx, myeloperoxidase, ANCA-associated vasculitis, antineutrophil cytoplasmic antibodies, vasculitis

## Abstract

**Case Description:**

A 16 year-old female who presented with initial ear, nose and throat manifestations who later progressed to severe renal disease, requiring hemodialysis after 11 months of unique laryngeal involvement.

**Clinical Findings:**

Unilateral vocal cord paralysis without other symptoms or signs, but with positive perinuclear anti-neutrophil cytoplasmic antibodies (ANCA) and anti-myeloperoxidase autoantibodies, followed an unfavorable course months later with rapidly progressive glomerulonephritis. Renal biopsy confirmed an ANCA-associated vasculitis. She was diagnosed with microscopic polyangiitis.

**Treatment and Outcome:**

High-dose glucocorticoids, intravenous cyclophosphamide, plasma exchange and finally, hemodialysis and renal transplantation.

**Clinical Relevance:**

In contrast to granulomatosis with polyangiitis (Wegener), ear, nose and throat manifestations in microscopic polyangiitis are uncommon, while involvement of the lungs and kidneys are usual. We present a case with an isolated rare involvement, which progressed to severe disease. This atypical case warns about laryngeal symptoms as initial manifestation of an anti-myeloperoxidase positive systemic vasculitides, and emphasizes the relevance of close observation when unexplained isolated conditions with accompanying evidence of autoimmunity, in this case high levels of specific autoantibodies, are present.

## Resumen

**Descripción del caso:**

Una mujer de 16 años se presentó inicialmente con manifestaciones otorrinolaringológicas y posteriormente progresó hacia enfermedad renal grave, requiriendo hemodiálisis después de 11 meses de tener exclusivamente afección laríngea.

**Hallazgos clínicos:**

parálisis de cuerda vocal unilateral sin otros síntomas ni signos, pero con autoanticuerpos anticitoplasma de neutrófilo (ANCA) con patrón perinuclear y especificidad contra mieloperoxidasa, siguiendo un curso desfavorable meses después con desarrollo de glomerulonefritis rápidamente progresiva. La biopsia renal confirmó una vasculitis asociada con ANCA (VAA). Se diagnosticó entonces como poliangitis microscópica.

**Tratamiento y desenlace:**

Glucocorticoides a dosis altas, ciclofosfamida endovenosa, recambio plasmático y finalmente, hemodiálisis y transplante renal.

**Relevancia clínica:**

en contraste con la granulomatosis con poliangitis (Wegener), las manifestaciones otorrinolaringológicas en poliangitis microscópica son poco comunes, mientras que la afección pulmonar y renal es común. Presentamos un caso con afección inusual aislaea, que progresó a enfermedad grave. Este caso atípico enfatiza sobre los síntomas laríngeos como manifestación inicial de una vasculitis antimieloperoxidasa positiva, y subraya la relevancia de una estrecha observación cuando condiciones aisladas inexplicables, que como en este caso se acompañan de evidencia de autoinmunidad manifestado por presencia de niveles altos de autoanticuerpos, se presentan para su atención.

## Introduction

Anti-neutrophil cytoplasmic antibodies (ANCA) are autoantibodies specific for antigens located in the cytoplasmic granules of neutrophils and lysosomes of monocytes. The ANCA-associated vasculitides (AAV) are multisystem diseases associated with autoantibodies that target myeloperoxidase (MPO-ANCA) and proteinase 3 (PR3-ANCA)^1^. The major clinicopathologic variants of AAV are microscopic polyangiitis (MPA), granulomatosis with polyangiitis (GPA), and eosinophilic granulomatosis with polyangiitis (EGPA) ^1^. 

Microscopic polyangiitis is a necrotizing vasculitis, with few or no immune deposits, predominantly affecting small vessels, i.e., capillaries, venules, or arterioles. Granulomatous inflammation is absent, differentiating it from GPA^1^. Patients typically present with fever, arthralgias or weight loss (61-78%), cutaneous manifestations (30-62%, mostly leukocytoclastic angiitis), peripheral neuropathy (37-72%), pulmonary disease (25-55%, capillaritis reported in 12-55%) and most importantly, renal involvement, characterized by crescentic necrotizing glomerulonephritis (80-100%) ^2^. Ear, nose and throat (ENT) symptoms are not common in MPA, and sometimes have been erroneously considered as an exclusive manifestation of GPA. ENT involvement may be an early sign of AAV; its presence has been associated with preserved renal function at disease onset, better long-term prognosis and improved survival ^1^.

Here, we present a MPA case with sole laryngeal involvement as initial manifestation, that later progressed to severe disease with rapidly progressive glomerulonephritis. 

## Case description

A 16 year-old female with a six-months history of dry cough and dyspnea was previously treated elsewhere with suspicion of infection, gastroesophageal reflux disease, asthma and allergy without improvement after several treatments. On arrival to our centre, these causes were sought and thoroughly discarded. The past medical history and physical examination were unremarkable. 

A fibronasolaryngoscopy showed arythenoidal and vocal cord edema (ventricular bands) with incomplete left vocal cord abduction. Diagnostic investigation for vocal cord paralysis included a head, neck and chest CT scan, excluding lymphadenopathy, malignancy or aneurysms. Flexible bronchoscopy did not show any lesions suspicious of neoplastic or granulomatous origin. There was no clinical evidence of degenerative neural disorders. The patient had no history of thoracic surgery, previous endotracheal intubation or cervical traumatisms. As airway disease is a well-described feature of GPA, ANCA by both methods (indirect immunofluorescence and antigen-specific ELISA, both by Euroimmun AG, Lübeck, Germany) were performed, with the following results: P-ANCA 1:20, MPO-ANCA 128 U/mL (normal <20), negative PR3-ANCA. Other relevant laboratory tests included serum creatinine (S-Cr) 0.73 mg/dL (64.5 μmol/L), C-reactive protein (C-RP) 1.22 mg/dL (normal <0.8), and haemoglobin (Hb) 12.7 g/dL. After careful search of potential conditions that could have explained her main symptoms, and in the absence of systemic involvement, the patient was labeled as having an "*MPO-ANCA positive isolated vocal cord paralysis*" and was sent to vocal rehabilitation with regular follow-up. Five months later, she presented with one-week history of vomit and distal legs edema; she was pale, with normal blood pressure, S-Cr 10.6 mg/dL (937 μmol/L), C-RP 5.88 mg/dL, Hb 9.7 g/dL. Urine sediment showed countless dysmorphic erythrocytes and granular casts. With the suspicion of acute kidney involvement associated with her MPO-ANCA positive status, treatment was started with 1 g methylprednisolone pulses, IV-cyclophosphamide (IV-CYC) (400 mg), hemodialysis and plasmapheresis, the latter stopped after 5 exchanges when renal biopsy showed pauciimmune necrotizing glomerulonephritis with global sclerosis in 90% of glomerulii. IV-CYC was continued monthly until complete remission (6x 400 mg pulses). Glucocorticoids were tapered until withdrawal and she remained in remission with azathioprine 50 mg qd and hemodialysis 3 times/week. Three years after the onset of renal failure, she received a renal graft (cadaveric donor) with optimal results. She is currently asymptomatic; S-Cr is 0.88 mg/dL (77.8 μmol/L), with normal urinalysis, blood cell count and acute phase reactants. She takes tacrolimus 3 mg bid, mycophenolate mofetil 500 mg tid and prednisone 5 mg qd. She improved after vocal rehabilitation and immunosuppressive treatment, with mild vocal cord paralysis as sequelae. The absence of granulomatous inflammation or other surrogate markers of GPA in addition to the presence of MPO-ANCA led us to establish MPA as the final diagnosis in this case.

## Discussion

ENT involvement is uncommon in MPA, described in 2-20% of patients in large series ^2^ ^-^ ^4^. These abnormalities accompany other disease manifestations at onset (16% of cases) or during relapses (3%) ^2^ ^-^ ^5^. They include sinusitis (6-9%), epistaxis (6-15%), unspecific rhinitis, polyps, sore throat (9-15%), mouth ulcers (21%) and deafness due to inner ear involvement (3%) ^2^ ^-^ ^4^. These otorhinolaryngological manifestations are usually mild, which contrast with the severe, infiltrative, and destructive nature of lesions usually observed in GPA. 

Two similar previous cases have been reported. In the first, as part of a series of 30 MPA patients, unspecified ENT involvement progressed to severe pulmonary-renal disease within two years ^6^. The second was a 74 years-old woman with granulomas in the vocal cords as initial manifestation of GPA. The disease did not evolve into systemic form ^7^. Vocal cord paralysis has also been reported in polyarteritis nodosa, Behcet's disease, rheumatoid arthritis and eosinophilic granulomatosis with polyangiitis, although never as an initial isolated manifestation ^8^ ^,^ ^9^. In these cases, as in ours, glucocorticoid administration improved vocal cord function.

Why our patient progressed from isolated laryngeal involvement to severe full-blown vasculitis is unknown. MPA may present acutely, with life-threatening manifestations associated with widespread multisystemic disease or may have an indolent course that lasts for weeks to months without evidence of specific organ involvement. In previous series, the interval between initial symptoms and MPA diagnosis ranged between 14 days to 7.46 years, with an average of 3.7 months to 2.6 years ^10^ ^,^ ^11^. This long lapse clearly illustrates the difficulty faced in recognizing MPA, particularly when disease onset includes solely constitutional symptoms, upper respiratory tract disease, or single-organ involvement ^2^ ^,^ ^7^. Regarding the latter, a renal-limited form of AAV, typically associated with MPO-ANCA and characterized by the absence of general symptoms and extrarenal vasculitis is usually considered part of the MPA spectrum, as its histopathologic features are indistinguishable from those found in systemic widespread MPA ^12^. Some patients who initially have this limited form may subsequently develop full-blown, more easily identifiable MPA ^11^.

As discrimination between major clinicopathological variants of AAV (GPA, MPA, EGPA) may be difficult, classification based in autoantibody specificity has been proposed. Categorization of cases as MPO-ANCA or PR3-ANCA seems to predict more accurately the response to therapy, long-term outcome, or relapse propensity rather than the clinical phenotypes of MPA or GPA ^13^. In this sense, our patient may be suffering from a MPO-ANCA-associated vasculitis with ENT and renal disease.

Finally, although the link between vocal cord paralysis and the occurrence 11 months later of severe MPO-ANCA disease cannot be definitively established as causal, the lack of evidence for an alternative origin of the laryngeal disease, the presence of MPO-ANCA from the start, the improvement associated with glucocorticoids and immunosuppressants, and the long-term follow-up confirming the diagnosis of MPA, lead us to believe that the most likely explanation of the cordal paralysis was related to involvement of the recurrent laryngeal nerve by MPA.

## Conclusion

To our best knowledge, this is the first case presenting with isolated vocal cord paralysis as initial manifestation of severe MPO-ANCA positive systemic vasculitis. In absence of more common causes of laryngeal disease, an unexplained etiology and the presence of positive ANCA testing, regular clinical and laboratory follow-up is advised in order to detect early, non-respiratory tract, serious and potentially irreversible organ involvement.
